# Seedling-Stage Responses of *Lumnitzera littorea* to Substrate Type and Salinity-Structured Irrigation Regimes in Can Gio, Vietnam

**DOI:** 10.3390/plants15111734

**Published:** 2026-06-03

**Authors:** Duc-Hoan Huynh, The-Kiet Bui-Nguyen, Ngoc-Hiep Dang, Thi-Phuong-Linh Nguyen, Thi-Thu-Thao Luong, Hoang-Dung Tran

**Affiliations:** 1 Management Board of Protection and Special-Use Forests of Ho Chi Minh City, 176 Hai Ba Trung Street, Tan Dinh Ward, Ho Chi Minh City 71088, Vietnam; joankietthe@gmail.com (T.-K.B.-N.); hqt.ngochiep93@gmail.com (N.-H.D.); ntplinh1709@gmail.com (T.-P.-L.N.); luongthithuthaobau99@gmail.com (T.-T.-T.L.); 2 Faculty of Biology and Environment, Ho Chi Minh City University of Industry and Trade (HUIT), 140 Le Trong Tan Street, Tay Thanh Ward, Ho Chi Minh City 72009, Vietnam; dungth@huit.edu.vn

**Keywords:** Can Gio, ex situ propagation, irrigation salinity, mangrove nursery, planting stock, substrate

## Abstract

Reliable nursery production is essential for producing ex situ planting stock of the locally threatened mangrove *Lumnitzera littorea* in Can Gio, Vietnam. We evaluated 12-month seedling performance using two nursery substrates—CTI (topsoil) and CTII (mixed substrate)—and salinity-structured irrigation regimes: C, a dynamic river/tidal water plus freshwater reference regime and seven fixed-salinity treatments (E1–E7: 0, 10, 15, 20, 25, 30, and 35‰). Each substrate–regime combination comprised three replicate cells of 40 seedlings (*N* = 1920). The primary endpoints were month 12 survival (SR12), seedling height among survivors (H12), and root collar diameter among survivors (Do12). Statistical inference was confined to E1–E7; C was retained as a descriptive operational reference. For E1–E7, substrate and regime significantly affected SR12 and H12, whereas Do12 varied by regime but not by substrate; no substrate × regime interaction was detected. SR12 declined sharply at 30–35‰, especially under CTI, and the high-salinity H12 and Do12 estimates were based on few survivors. CTII outperformed topsoil alone, particularly for survival and survivor-conditioned height. The findings support conservative nursery guidance for Can Gio: use a mixed substrate and avoid sustained high fixed-salinity irrigation, without extending the inference to a species-wide salinity optimum or post-planting field performance.

## 1. Introduction

Conserving *Lumnitzera littorea* in Vietnam depends on protecting remnant stands and producing reliable nursery stock. In Can Gio, where the species is rare and locally threatened, earlier Vietnamese studies reported practical difficulties in germination, early growth, and nursery handling [1,2,3]. The problem is also relevant beyond Can Gio because restoration and ex situ conservation programs often rely on a limited set of common, easily propagated mangrove taxa, whereas locally rare or less commonly propagated species may fail if their nursery requirements are assumed to match those of better studied species. Previous work on *L. littorea* has indicated that seed treatment, light, salinity, and early nursery conditions can affect seedling performance [1,2,3,4,5]. Together, these studies provide a local foundation, but they also leave a production gap: nurseries need treatment-level evidence linked to planting stock production, not only information about the species’ occurrence or conservation status in natural mangrove habitats.

Mangrove seedlings establish in physically demanding coastal environments where salinity, inundation, light, sediment condition, and post-germination stress can constrain early survival and growth. Comparative studies show that these responses differ among species, developmental stages, and experimental settings [6,7,8,9,10]. From a nursery perspective, the key question is therefore not whether a mangrove species is halophytic in a broad ecological sense, but whether a particular production regime can retain sufficient seedlings and support adequate growth over a defined nursery cycle. Survival, height, and root collar diameter are appropriate production endpoints because they describe both seedling availability and the condition of surviving planting stock [11,12,13,14,15]. 

In practical nursery production, irrigation salinity and rooting medium are two factors that managers can adjust directly. In estuarine nurseries such as Can Gio, irrigation water may come from river or tidal sources, freshwater supplementation, or prepared fixed-salinity solutions; the salinity experienced by seedlings in the nursery may therefore differ from the salinity of natural stands. The rooting medium can further influence seedling performance by affecting moisture retention, aeration, organic matter, and root zone conditions. Testing substrate type and assigned irrigation salinity under nursery regimes therefore addresses an operational production question. It does not define the natural salinity envelope of *L. littorea* or predict post-planting field performance.

Against this background, we framed this study as an original nursery experiment on seedling-stage responses of *L. littorea* under Can Gio production conditions. We evaluated the effects of substrate type and salinity-structured irrigation regime on three primary month 12 endpoints: survival rate (SR12), seedling height among survivors (H12), and root collar diameter among survivors (Do12). Our objective was to test these treatment effects and translate the results into conservative, locally relevant nursery guidance for ex situ planting stock production in Can Gio. The study was not designed to define a species-wide salinity optimum, characterize the in situ salinity range of natural *L. littorea* stands, or predict post-planting field performance.

## 2. Results

### 2.1. Experimental Design and Month 12 Accounting

**Figure 1:**
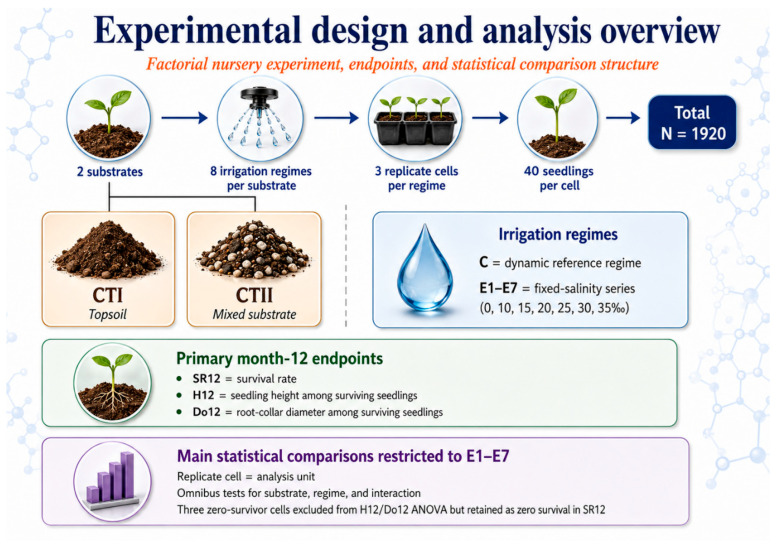
Nursery design and analysis framework. The experiment included two substrates, eight irrigation regimes per substrate, three replicate cells for each substrate–regime combination, and 40 seedlings per replicate cell (total *N* = 1920). C was retained as a descriptive dynamic reference regime, whereas E1–E7 formed the fixed-salinity series used for the main statistical comparisons. Primary month 12 endpoints were SR12, H12, and Do12; H12 and Do12 were evaluated among surviving seedlings only.

Figure 1 summarizes the experimental structure. Table 1 presents the main month 12 results and the primary endpoint summaries used to interpret treatment performance. For each irrigation regime, it reports the full SR12 denominator and the survivor-conditioned H12 and Do12 summaries under CTI and CTII, including contributing survivor counts and replicate cell coverage. Table S1 provides supporting replicate-level accounting and calculation details.

### 2.2. Survival at Month 12

Under the dynamic reference regime C, SR12 was 78/120 (65.0%) in CTI and 92/120 (76.7%) in CTII (Table 1). Because C had no fixed-salinity value, these values describe performance under the operational nursery routine rather than a position on the E1–E7 gradient. Within the fixed-salinity series, survival grouped into three broad zones rather than following a smooth monotonic decline. In CTI, SR12 remained 47.5–56.7% at E1–E2 (0–10‰), fell to approximately one-third at E3–E4 (32.5–33.3%), and then declined sharply at E5–E7 (16.7%, 7.5%, and 1.7%). In CTII, SR12 was highest at E1–E2 (71.7–76.7%), remained 45.0% at E3, fell at E4 (15.8%), and remained reduced at E5–E7 (32.5%, 20.8%, and 12.5%). Although the CTII pattern was not strictly monotonic, the clearest decline in nursery performance occurred toward the upper-middle and high-salinity ranges, especially at 30–35‰.

For E1–E7, replicate cell analysis showed significant effects of substrate (*p* = 0.015) and fixed-salinity regime (*p* < 0.001) on SR12, with no significant substrate × regime interaction (*p* = 0.271; Table 2). These results support overall substrate and regime effects on survival, but they do not support pairwise thresholds or substrate-specific response curves. Descriptively, CTII retained higher SR12 than CTI in six of the seven fixed-salinity regimes, with margins of 10.8–29.2 percentage points; E4 was the only exception. This pattern suggests a broad substrate-related survival advantage under CTII, but the nonsignificant interaction argues against overstating treatment-specific separation across salinity levels.

**Figure 2:**
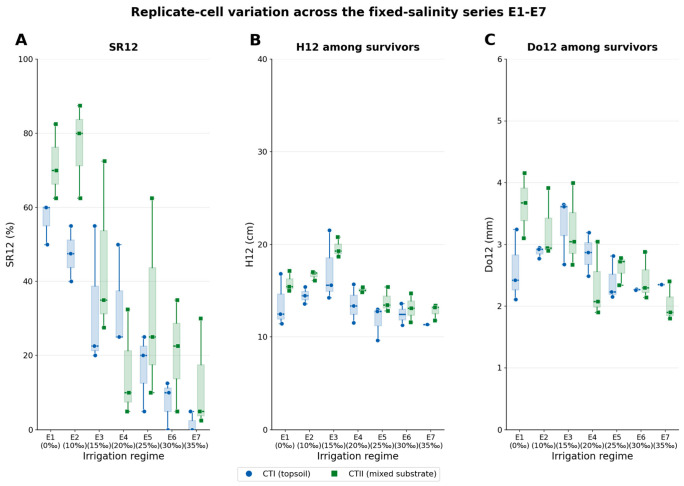
Replicate cell variation in month 12 responses across the fixed-salinity series E1–E7 under CTI (topsoil) and CTII (mixed substrate). (**A**) SR12, shown as replicate cell survival percentage. (**B**) H12, shown as replicate cell mean height of surviving seedlings. (**C**) Do12, shown as replicate cell mean root collar diameter of surviving seedlings. Boxes and whiskers summarize the available replicate cell values within each substrate–regime combination, and overlaid points show individual replicate cells. H12 and Do12 were calculated only for replicate cells with surviving seedlings; zero-survivor cells were therefore excluded from these survivor-conditioned endpoints. Figure 2 is restricted to the fixed-salinity series E1–E7 because C was retained as a descriptive dynamic reference regime rather than a fixed point in the salinity gradient.

Figure 2 displays the corresponding month 12 replicate cell variation across the fixed-salinity series, including SR12 and survivor-conditioned H12 and Do12 under CTI and CTII.

### 2.3. Height at Month 12 Among Survivors

H12 was survivor-conditioned and should be interpreted in the survival context shown in Table 1. Under the dynamic reference regime C, H12 was 22.90 cm in CTI and 23.11 cm in CTII. Across the fixed-salinity treatments, the highest descriptive H12 values occurred at E3 in both substrates: 18.60 cm in CTI and 19.20 cm in CTII. CTII maintained higher H12 than CTI at every fixed salinity, with descriptive differences of 0.6–2.3 cm across E1–E7. However, this height advantage was smaller than the survival contrast and did not offset the production constraint imposed by mortality at high salinity. At E5–E7, mean H12 remained measurable at 11.30–14.50 cm, but these values came from progressively smaller survivor sets rather than from the original full cohorts assigned to those regimes.

For E1–E7, replicate cell analysis showed significant effects of substrate (*p* = 0.0036) and fixed-salinity regime (*p* < 0.001) on H12, with no significant substrate × regime interaction (*p* = 0.987; Table 2). The sample sizes in Table 1 explain why high-salinity H12 means should be interpreted with caution: only nine CTI seedlings contributed at E6 and only two at E7, with CTI–E7 represented by a single contributing replicate cell. Thus, H12 at 30–35‰ describes height among surviving seedlings, not treatment-level production performance. The measurable height of the few surviving seedlings does not offset the severe survival loss at the high-salinity end.

### 2.4. Root Collar Diameter at Month 12 Among Survivors

Do12 was also survivor-conditioned and is reported with separate contributing counts in Table 1. Under the dynamic reference regime C, Do12 reached 4.40 mm in CTI and 4.86 mm in CTII. Across E1–E7, diameter did not track survival or height directly. CTI reached its highest descriptive Do12 at E3 (3.43 mm), whereas CTII was highest at E1 (3.67 mm) and remained similar at E3 (3.48 mm). At the high-salinity end, Do12 was consistently lower, falling to 2.27–2.35 mm in CTI and 2.29–2.60 mm in CTII at E6–E7. This narrower diameter range, together with the lack of a supported overall substrate effect, indicates that Do12 responded to the fixed-salinity series but did not indicate the same treatment pattern as survival and height.

At the replicate cell level for E1–E7, fixed-salinity regime significantly affected Do12 (*p* = 0.0024), whereas substrate (*p* = 0.284) and substrate × regime (*p* = 0.163) were not significant (Table 2). Thus, unlike SR12 and H12, root collar diameter did not show a statistically supported overall advantage of CTII at month 12. As with H12, Do12 at 30–35‰ was conditional on survival. These high-salinity means describe only the remaining survivors and should not be interpreted as evidence that the original treatment cohorts maintained acceptable planting stock performance.

### 2.5. Supporting Outcomes

Supporting variables, including total and mean monthly increments, followed the same broad pattern as the primary endpoints. Height- and diameter-related increments were generally greater in the mixed substrate than in topsoil alone at lower and intermediate salinities, whereas the high-salinity end of the fixed series showed clear deterioration in nursery performance. These outcomes are consistent with the main production-level interpretation: CTII improved survival and height margins under this nursery setup, while 30–35‰ remained unfavorable for planting stock production. Because the supporting outcomes do not change the core inference based on SR12, H12, and Do12, they are presented in the Supplementary Material rather than expanded upon in the main text.

**Figure 3:**
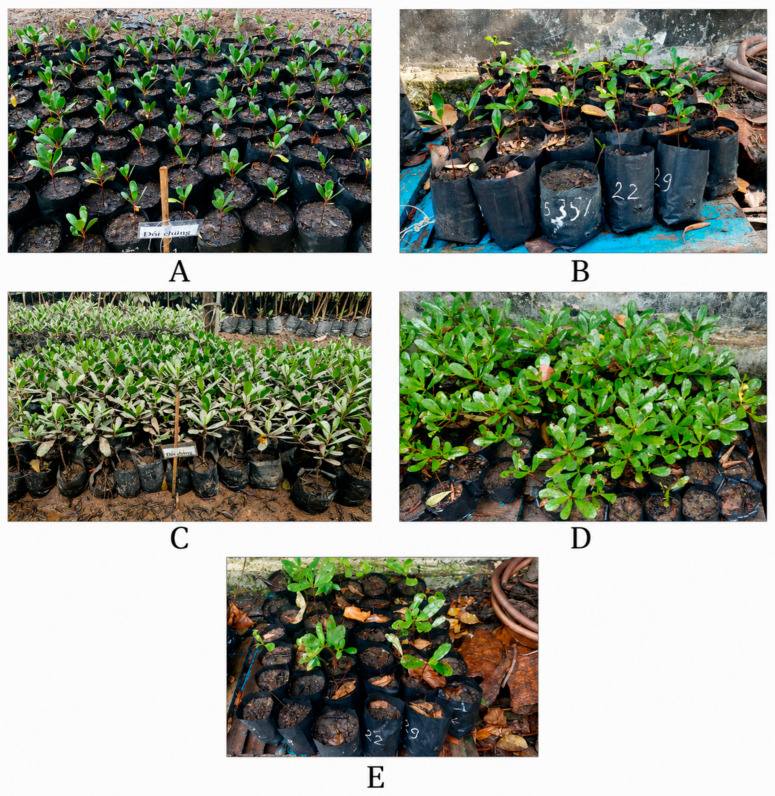
Representative nursery appearance of *Lumnitzera littorea* seedlings under selected irrigation regimes and time points. (**A**) Reference regime C at month 3 after initiation of the assigned irrigation regimes; (**B**) fixed 15‰ regime E3 at month 3; (**C**) reference regime C at month 11; (**D**) fixed 15‰ regime E3 at month 11; and (**E**) fixed 35‰ regime E7 at month 11. Seedlings were transplanted when they had reached the two-true-leaf stage after approximately three months of germination. True-leaf counts at month 3 and month 11; photo time points were not recorded as quantitative endpoints; the plate is therefore used as qualitative visual documentation of nursery condition and should not be interpreted as an additional statistical comparison. Numbers and salinity labels visible on nursery bags or wooden stakes are original nursery tracking marks used to identify replicate cells or assigned irrigation regimes during monitoring; they are retained in the photographs only as field labels and are not additional analytical variables.

Figure 3 provides representative seedling images from the nursery trial and links the quantitative endpoint patterns with visible differences in planting stock condition across selected regimes and time points. Figure 3A,B were taken at month 3 after the initiation of the assigned irrigation regimes, whereas Figure 3C–E were taken at month 11, one month before the final month 12 endpoint assessment. At transplanting, seedlings had reached the two-true-leaf stage after approximately three months of germination. True-leaf number at the month 3 and month 11 photo time points was not recorded as a quantitative endpoint. The plate therefore documents visible nursery condition and foliage development qualitatively, rather than serving as a counted leaf stage dataset or an additional statistical comparison.

## 3. Discussion

The central finding is that nursery-stage performance of *Lumnitzera littorea* seedlings varied with salinity-structured irrigation regime and, for survival and height, with substrate type. This evidence is best interpreted in the context of Can Gio nursery management for ex situ planting stock production, not as a characterization of the in situ salinity range of natural *L. littorea* stands. Within this nursery setting, survival remained the most direct production criterion because H12 and Do12 were measured only among survivors. This framing is consistent with earlier Vietnamese work that treated *L. littorea* propagation as a nursery problem rather than as a basis for generalized ecological tolerance claims [1,4,5].

Across the fixed-salinity series, seedling performance was stronger at lower to moderate salinity and declined markedly at 30–35‰. For nursery production, this distinction is important: a treatment that leaves only a small survivor pool is not operationally acceptable even if the remaining seedlings still have measurable height or diameter. This pattern is in accord with the mangrove seedling literature showing that high salinity can constrain early establishment and growth, although salinity ranges associated with better performance differ among species and experimental settings [6,7,8,9,10]. In the present dataset, the high-salinity effect was clearest in survival: CTI fell to 7.5% at 30‰ and 1.7% at 35‰, whereas CTII fell to 20.8% and 12.5% at the same salinities. Height and diameter among survivors did not follow survival exactly, but planting stock production first requires that enough seedlings remain alive. The results therefore support avoiding the upper fixed-salinity range in this Can Gio nursery setting, without defining a species-wide salinity optimum for *L. littorea*.

The response pattern is consistent with broader mangrove seedling studies showing that salinity effects are species-, stage-, and context-dependent and often interact with other growing conditions. Dangremond et al. [16] compared seedlings of four mangrove species along light and salinity gradients and found that survival and growth responses differed among taxa, with high-salinity conditions reducing performance in several species. López-Hoffman et al. [17,18] likewise showed that salinity and light can jointly shape mangrove seedling photosynthesis, growth, and survivorship. Krauss and Ball [19] cautioned against treating mangroves as uniformly salt-loving plants, and Bompy et al. [20] showed that soil salinity fluctuation can affect seedling growth and physiology. These comparisons support the framing used here: the Can Gio experiment identifies production-relevant responses of *L. littorea* seedlings under assigned nursery irrigation regimes, rather than a general physiological tolerance range for the species.

Budiadi et al. [13] offer a useful comparison. In their ex situ nursery study of *Avicennia marina*, seedlings were grown under several growth media and four salinity bands: 5‰, 5–15‰, 15–25‰, and 25–35‰. Survival was the strongest at 5–15‰, whereas higher salinity reduced survival. The present *L. littorea* experiment provides a similar directional warning over a longer 12-month nursery period and a wider fixed-salinity series: at 30–35‰, SR12 declined to 7.5% and 1.7% in CTI and to 20.8% and 12.5% in CTII. This comparison does not transfer an optimum from *A. marina* to *L. littorea*, as the two studies differed in species, salinity design, exposure duration, substrate composition, and endpoint structure. Rather, both studies show that ex situ mangrove seedling production can be compromised in upper salinity ranges and that nursery recommendations should remain species- and context-specific.

Zhou et al. [14] provide another useful comparison because their *Kandelia obovata* nursery study combined salinity, growth media, and genealogy while measuring mortality, height, diameter, and biomass-related responses. Together with Budiadi et al. [13], these studies show that salinity is applied within a nursery context shaped by the growing medium and seedling quality. Grossnickle and MacDonald [15] further emphasized that seedling performance depends on plant attributes that emerge from the production environment. The present experiment cannot rank substrate mechanisms, biomass allocation, genotype effects, or root zone processes because the physical and chemical properties of CTI and CTII were not directly quantified. Its contribution is narrower: CTII was empirically the more reliable medium for local *L. littorea* planting stock production, especially for survival and survivor-conditioned height, while the underlying mechanisms remain to be tested.

C remains useful as a practical reference, but only within a limited interpretive scope. This regime used river or tidal water supplemented with freshwater as needed, and its actual salinity range, temporal variation, and operational criteria for freshwater supplementation were not recorded. It therefore represented a workable nursery routine rather than a constant salinity treatment. The descriptive performance of C shows that this operational regime can maintain seedlings under the local setup, but it cannot be inserted into the fixed gradient, compared statistically with E1–E7, or interpreted as evidence of acclimation to a specific salinity level.

The substrate effect should also be read in the nursery production context. CTII generally maintained stronger survival and greater survivor-conditioned height than CTI, suggesting that the mixed substrate was the more suitable nursery medium in this experiment. This finding is consistent with broader nursery literature showing that early mangrove seedling performance is shaped not only by water salinity but also by rooting medium properties, sediment context, organic amendments, and seedling attributes [11,12,13,14,15]. At the same time, the nonsignificant substrate × regime interaction prevents a substrate-specific salinity threshold from being inferred. A cautious management interpretation is that CTII improved overall nursery reliability, while the warning against sustained irrigation at the upper fixed-salinity range remained broadly applicable across both substrates within the tested design.

Accordingly, the replicate cell variation shown in Figure 2 supports a production-level interpretation—CTII was generally more reliable and the upper fixed-salinity range was unfavorable—while the limitations below define what the experiment cannot infer.

Several limitations define the scope of inference. First, H12 and Do12 are survivor-conditioned and must be interpreted alongside SR12 and contributing sample sizes, especially at high salinity. Second, C was dynamic and operational and therefore cannot be inserted into the fixed-salinity gradient or used to estimate a salinity optimum. Third, irrigation was applied under a common nursery routine within assigned regimes, but continuous records of irrigation frequency, irrigation volume, freshwater supplementation, EC, and water balance were not retained; the experiment therefore supports comparison of concurrent substrate × regime treatments, not reconstruction of the realized cumulative salt or water dose. Finally, physiological, biochemical, biomass allocation, water balance, and post-planting field responses were not measured; such processes are important in mangrove responses to salinity [19,20]. The findings should therefore be applied as nursery-stage guidance for ex situ planting stock production in similar Can Gio production settings, not as evidence for whole-species salinity physiology, restoration zoning, or field establishment success.

## 4. Materials and Methods

### 4.1. Study Site

The trial was conducted at the nursery of the Management Board of Protection and Special-use Forests of Ho Chi Minh City, located in compartment 1, plot 25, sub-compartment 10b, in the Can Gio Mangrove Biosphere Reserve. The experimental area was established on elevated nursery ground that was not directly inundated by the tide during the study period.

### 4.2. Plant Material and Substrates

After approximately three months of germination, seedlings that had reached the two-true-leaf transplanting stage were transferred into 7 cm × 10 cm polymer bags. During transplanting, approximately one-fifth to one-quarter of the basal stem segment, up to the root collar, was buried in the substrate. True-leaf number after transplanting was not used as a measured endpoint; subsequent seedling condition was followed through survival, height, root collar diameter, and representative nursery photographs.

Two nursery substrates were tested. CTI consisted of topsoil collected at the nursery. CTII was a mixed substrate containing 40% topsoil, 20% sand, and 40% organic amendment mixture; the organic fraction was composed equally of rice husk, rice husk ash, coconut coir, and cattle manure. In practice, CTII represented a lighter and more structured nursery medium than topsoil alone.

### 4.3. Experimental Design and Irrigation Regimes

The experiment used a 2 × 8 × 3 × 40 nursery design: two substrates, eight irrigation regimes per substrate, three replicate cells for each substrate–regime combination, and 40 seedlings per replicate cell. This design yielded 120 seedlings per substrate–regime combination, 960 seedlings per substrate, and 1920 seedlings overall. The 40-seedling replicate cell was the unit of inference; individual seedlings were observational units. Figure 1 summarizes the design. Table S1 provides replicate-level accounting and calculation details, whereas Table 1 reports the main month 12 primary endpoints.

After transplanting, seedlings were monitored for 10 days, and individuals lost during handling were removed before the trial began. All treatments were maintained concurrently under the same nursery background conditions. Within each irrigation round, replicate cells received the assigned irrigation regime under the same nursery routine, so the experimental contrast was substrate type and irrigation salinity regime rather than differential watering intensity. Irrigation followed the assigned regimes, and fixed-salinity solutions were prepared, checked, and stored separately from the dynamic reference regime. The available operation records document treatment assignment, target salinity preparation, survival, height, and root collar diameter endpoints, but they do not retain a continuous log of irrigation frequency, irrigation volume, or detailed freshwater supplementation triggers for C. The results are therefore interpreted as responses to assigned nursery irrigation regimes with documented target salinity preparation, not as estimates of cumulative salt dose, leaching, or water balance.

The eight irrigation regimes were defined as follows: C, dynamic river/tidal water with freshwater supplementation as needed; E1, fixed freshwater (0‰); E2, fixed 10‰; E3, fixed 15‰; E4, fixed 20‰; E5, fixed 25‰; E6, fixed 30‰; and E7, fixed 35‰. Solutions for E2–E7 were prepared from nursery river water by dilution with domestic freshwater or by adding locally produced sea salt to reach the target salinity. Salinity was checked with an Atago Master-S28alpha salinity refractometer, and prepared solutions were stored in sealed 20 L containers under shade. The experiment was reported in parts per thousand (‰; ppt-equivalent). Electrical conductivity (EC, dS m^−1^) was not measured for the river/tidal water, freshwater source, or fixed treatments; EC-equivalent values are therefore not reported.

C served as a dynamic operational reference rather than a classical control. Its salinity varied over time with river or tidal water conditions and freshwater supplementation, and the actual salinity range and temporal pattern were not available. C was therefore kept separate from the fixed-salinity gradient in analysis and interpretation. It can describe operational feasibility under the local nursery routine, but it cannot be used for statistical comparison with E1–E7, gradient fitting, or estimation of a salinity optimum. The fixed-salinity treatments should therefore be interpreted as irrigation regimes structured by salinity, not as an assay of whole-species salinity physiology.

### 4.4. Measurements and Endpoint Definitions

Seedling height was measured every three months from the substrate surface to the apical meristem. Root collar diameter (Do) was measured every six months with a caliper. The nursery experiment lasted 12 months, from 1 November 2024 to 31 October 2025.

Three endpoints were designated as primary: survival rate at month 12 (SR12), seedling height at month 12 among surviving seedlings (H12), and root collar diameter at month 12 among surviving seedlings (Do12). Supporting variables included total height increment, total root collar diameter increment, and mean monthly increments. The Do/H index was not retained as a main text endpoint.

A seedling contributed to SR12 if it was recorded as alive at the final assessment. H12 and Do12 were calculated only for surviving seedlings with available month 12 measurements. Final height and root collar diameter records were matched to verified survivor records before month 12 growth summaries were calculated. Consequently, H12 and Do12 are survivor-conditioned outcomes; they were therefore interpreted together with SR12 rather than as standalone growth responses.

### 4.5. Data Preparation and Statistical Analysis

The analysis used the complete 2 × 8 × 3 × 40 experimental structure. Final month 12 height and root collar diameter values were matched to final survivor records before endpoint summaries were calculated. Three replicate cells had no survivors at month 12: CTI–E6 replicate 1, CTI–E7 replicate 1, and CTI–E7 replicate 2.

For statistical analysis, fixed-salinity treatments E1–E7 were evaluated at the replicate cell level, whereas the dynamic reference regime C was summarized descriptively. SR12 was analyzed as replicate cell survival percentage in a two-factor factorial ANOVA with substrate, fixed-salinity regime, and their interaction. H12 and Do12 were analyzed with the same factorial structure, using replicate cell means from cells with surviving seedlings. The three zero-survivor cells were excluded from the H12 and Do12 ANOVA but retained as zero-survival cells in the SR12 analysis. This approach follows the survivor-conditioned definition of H12 and Do12; it also means that high-salinity growth means describe only surviving seedlings and may reflect the remaining survivor subset rather than full treatment-level performance.

Only overall factorial tests for substrate, fixed-salinity regime, and substrate × regime interaction are reported; inference is therefore limited to those overall effects. No pairwise or post hoc salinity threshold claims are presented. The analyses were conducted in Python 3.13.5, using pandas 2.2.3 for data handling and statsmodels 0.14.6 for ANOVA; statistical significance was interpreted at α = 0.05.

## 5. Conclusions

This study evaluated how substrate type and salinity-structured irrigation regimes affected 12-month nursery-stage performance of *Lumnitzera littorea* seedlings in Can Gio. In the fixed-salinity series E1–E7, substrate and regime significantly affected SR12 and survivor-conditioned H12, whereas Do12 was affected by regime but not by substrate; no substrate × regime interaction was supported for any primary endpoint. For Can Gio ex situ nursery production, the clearest practical recommendation from the tested design is to use CTII, the mixed substrate, rather than topsoil alone. Where a fixed-salinity irrigation protocol is required, CTII–E2 (10‰) is the most defensible fixed-salinity candidate based on its descriptive balance of high SR12 and acceptable survivor-conditioned growth, while CTII–E3 (15‰) may be considered where slightly greater survivor-conditioned height is operationally valued but with lower survival. The dynamic C regime also remains a practical local reference when river/tidal water and freshwater supplementation can be managed consistently. Sustained high fixed-salinity irrigation at 30–35‰ (E6–E7) should be avoided for routine nursery production of *L. littorea* seedlings under comparable Can Gio conditions. These recommendations remain nursery-stage guidance only; field validation is required before any restoration, zoning, or post-planting performance inference.

## Figures and Tables

**Table 1:** Month 12 endpoint summaries by irrigation regime, with CTI and CTII shown as parallel substrate blocks.

Regime	Regime Definition	CTI: Topsoil					CTII: Mixed Substrate				
		SR12	H12 n Survivors (Replicate Cells)	H12 Mean ± SD (cm)	Do12 n Survivors (Replicate Cells)	Do12 Mean ± SD (mm)	SR12	H12 n Survivors (Replicate Cells)	H12 Mean ± SD (cm)	Do12 n Survivors (Replicate Cells)	Do12 Mean ± SD (mm)
C	Dynamic descriptive reference: river/tidal water + freshwater as needed	78/120 (65.0%)	78 (3/3)	22.90 ± 6.79	78 (3/3)	4.40 ± 1.66	92/120 (76.7%)	88 (3/3)	23.11 ± 2.94	91 (3/3)	4.86 ± 1.13
E1	Fixed salinity: 0‰	68/120 (56.7%)	68 (3/3)	13.60 ± 3.38	68 (3/3)	2.60 ± 0.77	86/120 (71.7%)	86 (3/3)	15.70 ± 3.10	87 (3/3)	3.67 ± 0.87
E2	Fixed salinity: 10‰	57/120 (47.5%)	57 (3/3)	14.40 ± 2.93	57 (3/3)	2.88 ± 0.78	92/120 (76.7%)	92 (3/3)	16.70 ± 2.49	92 (3/3)	3.30 ± 0.82
E3	Fixed salinity: 15‰	39/120 (32.5%)	39 (3/3)	18.60 ± 5.42	40 (3/3)	3.43 ± 1.19	54/120 (45.0%)	54 (3/3)	19.20 ± 2.85	54 (3/3)	3.48 ± 0.87
E4	Fixed salinity: 20‰	40/120 (33.3%)	40 (3/3)	13.48 ± 2.17	40 (3/3)	2.76 ± 0.67	19/120 (15.8%)	19 (3/3)	15.00 ± 1.50	19 (3/3)	2.72 ± 0.65
E5	Fixed salinity: 25‰	20/120 (16.7%)	20 (3/3)	12.50 ± 2.99	20 (3/3)	2.46 ± 0.95	39/120 (32.5%)	39 (3/3)	14.50 ± 2.22	39 (3/3)	2.66 ± 0.49
E6	Fixed salinity: 30‰	9/120 (7.5%)	9 (2/3)	12.30 ± 2.51	9 (2/3)	2.27 ± 0.44	25/120 (20.8%)	25 (3/3)	13.40 ± 2.66	23 (3/3)	2.60 ± 0.50
E7	Fixed salinity: 35‰	2/120 (1.7%)	2 (1/3)	11.30 ± 3.75	2 (1/3)	2.35 ± 0.35	15/120 (12.5%)	15 (3/3)	13.10 ± 2.08	14 (3/3)	2.29 ± 0.40

Note: SR12 is reported with the full denominator for each substrate–regime combination. H12 and Do12 are survivor-conditioned endpoints; the table therefore gives the contributing survivor count and replicate cell coverage for each endpoint. Because month 12 height and diameter records were verified separately, contributing counts may differ slightly between H12 and Do12 within the same regime. C is a dynamic descriptive reference regime based on river/tidal water with freshwater supplementation as needed. E1–E7 are fixed-salinity treatments at 0, 10, 15, 20, 25, 30, and 35‰, respectively. EC-equivalent values are not reported because conductivity was not measured or archived.

**Table 2:** Replicate cell factorial inference for the fixed-salinity treatments E1–E7, with *p*-values and effect status.

Endpoint	Analysis Unit	Analysis Set	Response/Model	Empty Cell Handling	Substrate Effect	Regime Effect	Substrate × Regime Effect
SR12	Replicate cell	E1–E7; 42 cells	Replicate cell survival proportion; two-factor factorial ANOVA	None	Significant (*p* = 0.015)	Significant (*p* < 0.001)	Not significant (*p* = 0.271)
H12 among survivors	Replicate cell with survivors	E1–E7; 39 non-empty cells	Mean height among survivors; two-factor factorial ANOVA	Three zero-survivor cells omitted	Significant (*p* = 0.0036)	Significant (*p* < 0.001)	Not significant (*p* = 0.987)
Do12 among survivors	Replicate cell with survivors	E1–E7; 39 non-empty cells	Mean root collar diameter among survivors; two-factor factorial ANOVA	Three zero-survivor cells omitted	Not significant (*p* = 0.284)	Significant (*p* = 0.0024)	Not significant (*p* = 0.163)

Note. Effect status is reported at α = 0.05. C was excluded from the main statistical comparisons and retained as a descriptive operational reference regime. No pairwise post hoc claims are reported in the main text. For H12 and Do12, zero-survivor cells were omitted because these endpoints were defined among surviving seedlings only.

## Data Availability

Additional underlying nursery records are available from the corresponding author upon reasonable request.

## Supplementary Materials

The following supporting information can be downloaded at: https://www.mdpi.com/article/10.3390/plants15111734/s1, Table S1: Month-12 accounting, replicate-level endpoint summaries, and supporting calculations; Table S2. Replicate cells with unequal H12 and Do12 contributing counts; Table S3. Zero-survivor replicate cells omitted from H12/Do12 factorial analysis; Table S4. Factorial comparison structure for the fixed-salinity treatments E1–E7; Table S5A. Height-related supporting outcomes by substrate × irrigation regime; Table S5B. Root-collar-diameter-related supporting outcomes by substrate × irrigation regime; Table S6. Replicate-cell survivor counts and survivor-conditioned means at month 12; Workbook S1: Supplementary workbook containing replicate-cell endpoint summaries, Figure2_plot_data, Extended_Replicates, and the workbook guide.
