# Supplementary material for: Seedling-Stage Responses of Lumnitzera littorea to Substrate Type and Salinity-Structured Irrigation Regimes in Can Gio, Vietnam
Source: Plants (Basel). 2026 Jun 3;15(11):1734. doi: 10.3390/plants15111734 (PMC13259365; doi:10.3390/plants15111734)
Supplement: Supplementary file 1 [file plants-15-01734-s001.zip › plants-4293280_supplementary_material.pdf]

Supplementary Material

Supplementary Material for: Nursery-stage responses of Lumnitzera littorea seedlings to substrate type and salinity-structured irrigation regimes in Can Gio, Vietnam

This Supplementary Material provides supporting tables for the experimental design, month-12 accounting, replicate-level reporting, and secondary outcomes accompanying the main manuscript.

Table S1. Design and month-12 accounting by substrate × irrigation regime.

Substrate	Regime	SR12 survivors	SR12 denominator	SR12 percent	H12 n survivors	H12 cells	H12 mean ± SD surv cm	Do12 n survivors	Do12 cells	Do12 mean ± SD surv mm
CTI	C	78	120	65.0	78	3	22.90 ± 6.79	78	3	4.40 ± 1.66
CTII	C	92	120	76.7	88	3	23.11 ± 2.94	91	3	4.86 ± 1.13
CTI	E1	68	120	56.7	68	3	13.60 ± 3.38	68	3	2.60 ± 0.77
CTII	E1	86	120	71.7	86	3	15.70 ± 3.10	87	3	3.67 ± 0.87
CTI	E2	57	120	47.5	57	3	14.40 ± 2.93	57	3	2.88 ± 0.78
CTII	E2	92	120	76.7	92	3	16.70 ± 2.49	92	3	3.30 ± 0.82
CTI	E3	39	120	32.5	39	3	18.60 ± 5.42	40	3	3.43 ± 1.19
CTII	E3	54	120	45.0	54	3	19.20 ± 2.85	54	3	3.48 ± 0.87
CTI	E4	40	120	33.3	40	3	13.48 ± 2.17	40	3	2.76 ± 0.67
CTII	E4	19	120	15.8	19	3	15.00 ± 1.50	19	3	2.72 ± 0.65
CTI	E5	20	120	16.7	20	3	12.50 ± 2.99	20	3	2.46 ± 0.95
CTII	E5	39	120	32.5	39	3	14.50 ± 2.22	39	3	2.66 ± 0.49
CTI	E6	9	120	7.5	9	2	12.30 ± 2.51	9	2	2.27 ± 0.44
CTII	E6	25	120	20.8	25	3	13.40 ± 2.66	23	3	2.60 ± 0.50
CTI	E7	2	120	1.7	2	1	11.30 ± 3.75	2	1	2.35 ± 0.35
CTII	E7	15	120	12.5	15	3	13.10 ± 2.08	14	3	2.29 ± 0.40

Note. SR12 is reported with its full denominator. H12 and Do12 are survivor-conditioned. Separate H12 and Do12 counts are retained because verification of the month-12 height and diameter sheets identified a small number of differing records, which resulted in unequal contributing counts in a few replicate cells.

Section S2. Note on differing H12 and Do12 contributing counts

Verification of the month-12 height and diameter sheets identified 10 differing seedling records. After data verification, unequal H12 and Do12 contributing counts remained in the following replicate cells:

Table S2. Replicate cells with unequal H12 and Do12 contributing counts.

Substrate	Regime	Replicate cell	H12 n survivors	Do12 n survivors	Difference H minus Do
CTII	C	2	27	30	-3
CTII	E1	2	28	29	-1
CTI	E3	3	22	23	-1
CTII	E6	2	9	7	2
CTII	E7	1	12	11	1

Interpretive note. Separate contributing counts for H12 and Do12 are therefore retained in the main manuscript instead of forcing a single shared survivor count for both endpoints.

Section S3. Note on the three zero-survivor replicate cells

Three replicate cells contributed no survivor-conditioned H12 or Do12 data at month 12 and were therefore omitted from the replicate-cell factorial ANOVA for H12 and Do12.

Table S3. Zero-survivor replicate cells omitted from H12/Do12 factorial analysis.

Substrate	Regime	Replicate cell	H12 n survivors	Do12 n survivors	Zero survivor H12 cell	Zero survivor Do12 cell
CTI	E6	1	0	0	Yes	Yes
CTI	E7	1	0	0	Yes	Yes
CTI	E7	2	0	0	Yes	Yes

Section S4. Replicate-cell comparison structure used in the main text

Table S4. Factorial comparison structure for the fixed-salinity treatments E1–E7.

Endpoint	Analysis set	Model / response	Empty-cell handling	Substrate p	Regime p	Substrate × regime p	Main-text inference
SR12	42 replicate cells from E1–E7	Two-factor factorial ANOVA on replicate-cell survival percentage	None	0.015	<0.001	0.271	Overall factorial tests only
H12 among survivors	39 non-empty replicate cells from E1–E7	Two-factor factorial ANOVA on replicate-cell mean height among survivors	Three zero-survivor cells omitted	0.0036	<0.001	0.987	Overall factorial tests only
Do12 among survivors	39 non-empty replicate cells from E1–E7	Two-factor factorial ANOVA on replicate-cell mean root-collar diameter among survivors	Three zero-survivor cells omitted	0.284	0.0024	0.163	Overall factorial tests only

Note. The dynamic reference regime C remained descriptive only and was excluded from the main statistical comparisons.

Section S5. Supporting outcomes moved out of the main text

Table S5A. Height-related supporting outcomes by substrate × irrigation regime.

Substrate	Regime	H support n	H support cells	Total H increment cm mean	Total H increment cm SD	Monthly H increment cm mean	Monthly H increment cm SD
CTI	C	78	3	17.25	6.56	1.44	0.55
CTII	C	88	3	17.58	2.85	1.47	0.24
CTI	E1	68	3	8.40	3.21	0.70	0.27
CTII	E1	86	3	10.79	2.68	0.90	0.22
CTI	E2	57	3	9.22	2.77	0.77	0.23
CTII	E2	92	3	11.27	2.32	0.94	0.19
CTI	E3	39	3	13.29	5.70	1.11	0.47
CTII	E3	54	3	13.76	2.72	1.15	0.23
CTI	E4	40	3	8.23	1.82	0.69	0.15
CTII	E4	19	3	9.63	1.70	0.80	0.14
CTI	E5	20	3	7.02	2.72	0.59	0.23
CTII	E5	39	3	9.91	2.10	0.83	0.18
CTI	E6	9	2	7.53	2.69	0.63	0.22
CTII	E6	25	3	8.15	2.41	0.68	0.20
CTI	E7	2	1	5.85	2.62	0.49	0.22
CTII	E7	15	3	7.81	2.22	0.65	0.18

Table S5B. Root-collar-diameter-related supporting outcomes by substrate × irrigation regime.

Substrate	Regime	Do support n	Do support cells	Total Do increment mm mean	Total Do increment mm SD	Monthly Do increment mm mean	Monthly Do increment mm SD
CTI	C	78	3	3.02	1.54	0.25	0.13
CTII	C	91	3	3.91	1.13	0.33	0.09
CTI	E1	68	3	1.58	0.75	0.13	0.06
CTII	E1	87	3	2.64	0.77	0.22	0.06
CTI	E2	57	3	1.82	0.80	0.15	0.07
CTII	E2	92	3	2.27	0.74	0.19	0.06
CTI	E3	40	3	2.28	1.09	0.19	0.09
CTII	E3	54	3	2.48	0.84	0.21	0.07
CTI	E4	40	3	1.71	0.59	0.14	0.05
CTII	E4	19	3	1.73	0.61	0.14	0.05
CTI	E5	20	3	1.30	0.96	0.11	0.08
CTII	E5	39	3	1.72	0.50	0.14	0.04
CTI	E6	9	2	1.32	0.31	0.11	0.03

Substrate	Regime	Do support n	Do support cells	Total Do increment mm mean	Total Do increment mm SD	Monthly Do increment mm mean	Monthly Do increment mm SD
CTII	E6	23	3	1.59	0.47	0.13	0.04
CTI	E7	2	1	1.35	0.35	0.11	0.03
CTII	E7	14	3	1.23	0.40	0.10	0.03

Note. These supporting outcomes do not change the main interpretive focus of the manuscript, which remains centered on SR12, H12, and Do12.

Section S6. Compact replicate-level appendix table

Table S6. Replicate-cell survivor counts and survivor-conditioned means at month 12.

Substrate	Regime	Replicate cell	H12 n survivors	H12 mean cm	Do12 n survivors	Do12 mean mm
CTI	C	1	24	24.38	24	4.29
CTI	C	2	25	22.71	25	4.54
CTI	C	3	29	21.60	29	4.37
CTII	C	1	31	23.60	31	4.71
CTII	C	2	27	23.48	30	5.19
CTII	C	3	30	22.24	30	4.69
CTI	E1	1	24	11.43	24	2.11
CTI	E1	2	24	16.82	24	3.25
CTI	E1	3	20	12.49	20	2.42
CTII	E1	1	33	15.00	33	4.16
CTII	E1	2	28	15.45	29	3.10
CTII	E1	3	25	17.14	25	3.67
CTI	E2	1	19	13.57	19	2.77
CTI	E2	2	16	15.41	16	2.94
CTI	E2	3	22	14.45	22	2.92
CTII	E2	1	35	16.91	35	3.92
CTII	E2	2	32	17.02	32	2.94
CTII	E2	3	25	16.11	25	2.90
CTI	E3	1	9	15.60	9	3.64
CTI	E3	2	8	14.26	8	2.67
CTI	E3	3	22	21.52	23	3.61
CTII	E3	1	29	18.70	29	4.00
CTII	E3	2	11	20.79	11	2.67
CTII	E3	3	14	19.30	14	3.04
CTI	E4	1	10	15.69	10	3.19
CTI	E4	2	10	11.53	10	2.87
CTI	E4	3	20	13.35	20	2.48

Substrate	Regime	Replicate cell	H12 n survivors	H12 mean cm	Do12 n survivors	Do12 mean mm
CTII	E4	1	13	15.05	13	3.05
CTII	E4	2	2	14.85	2	1.90
CTII	E4	3	4	15.38	4	2.08
CTI	E5	1	2	9.60	2	2.15
CTI	E5	2	8	13.00	8	2.81
CTI	E5	3	10	12.78	10	2.23
CTII	E5	1	4	13.48	4	2.72
CTII	E5	2	25	15.40	25	2.78
CTII	E5	3	10	12.83	10	2.34
CTI	E6	1	0		0	
CTI	E6	2	4	13.62	4	2.27
CTI	E6	3	5	11.26	5	2.26
CTII	E6	1	14	14.71	14	2.88
CTII	E6	2	9	11.61	7	2.14
CTII	E6	3	2	13.10	2	2.30
CTI	E7	1	0		0	
CTI	E7	2	0		0	
CTI	E7	3	2	11.35	2	2.35
CTII	E7	1	12	13.39	11	2.40
CTII	E7	2	1	13.20	1	1.80
CTII	E7	3	2	11.80	2	1.90

Note. The accompanying supplementary workbook provides a wider replicate-level summary, including total and monthly increment descriptors.
